# Multi-omics reveals glucose repression of citric acid catabolism in *Pichia kudriavzevii*

**DOI:** 10.1007/s00253-025-13590-3

**Published:** 2025-09-16

**Authors:** Yichao Cheng, Xinyi Wang, Di Wu, Yao Lu, Yi Qin, Yanlin Liu, Yanying Liang, Yuyang Song

**Affiliations:** 1 College of Enology, Northwest A&F University, Yangling, 712100 Shaanxi China; 2 Ningxia Helan Mountain’s East Foothill Wine Experiment and Demonstration Station of Northwest, A&F University, Yongning, 750104 Ningxia China

**Keywords:** *Pichia kudriavzevii*, Carbon catabolism repression (CCR), Citrate acid, Metabolomics, Transcriptomics

## Abstract

**Abstract:**

*Pichia kudriavzevii* is a widely used yeast in the wine industry that can degrade citric acid. However, this process can be hindered by the presence of glucose through a phenomenon called carbon catabolite repression (CCR). Herein, this study determined the underlying mechanism by examining the effects of glucose on *P. kudriavzevii*. Our findings indicated that glucose inhibited the reduction of citric acid and maintained elevated levels of fatty acids and glycerophospholipids. However, the inhibition of citric acid degradation under glucose addition was related to the retarded accumulation of metabolites involved in the biosynthesis of antibiotics, propanoate metabolism, microbial metabolism in diverse environments, C5-branched dibasic acid metabolism, and metabolic pathways in diverse environments. Additionally, the integrated data revealed that citrate catabolism of *P. kudriavzevii* was remarkably repressed in response to glucose by regulating glycerophospholipid metabolism, carbon metabolism and the biosynthesis pathways of secondary metabolites. Further investigations indicated that the increase of fatty acids (e.g., alpha-linolenic and arachidic) and glycerophospholipids (e.g., dihydroxyacetone phosphate and glycerophosphocholine) under glucose addition was related to the up-regulated *GPD1*, *PISD, HIS1* and *RPIA* gene expressions in glycerophospholipid metabolism and the down-regulated *FBP1*, *MDH*, *IDH3*, *ICL1*, *ACL* and *JEN1* gene expressions in carbon metabolism and the biosynthesis pathways of secondary metabolites. Meantime, glucose regulated the expression of transcription factors (e.g., *MIG1* and *GCN4*) associated with three pathways, which were crucial genes of CCR regulatory networks. Overall, we uncovered the metabolic regulatory network through which CCR inhibits citric acid utilization in *P. kudriavzevii*.

**Key points:**

• *Metabolic changes of P. kudriavzevii cells responding to carbon sources were observed*

• *Potential genes regulating citric acid degradation contributing to CCR were screened*

• *The inhibition of citric acid degradation is due to changes in the regulatory network*

**Graphical Abstract:**

**Figure:**
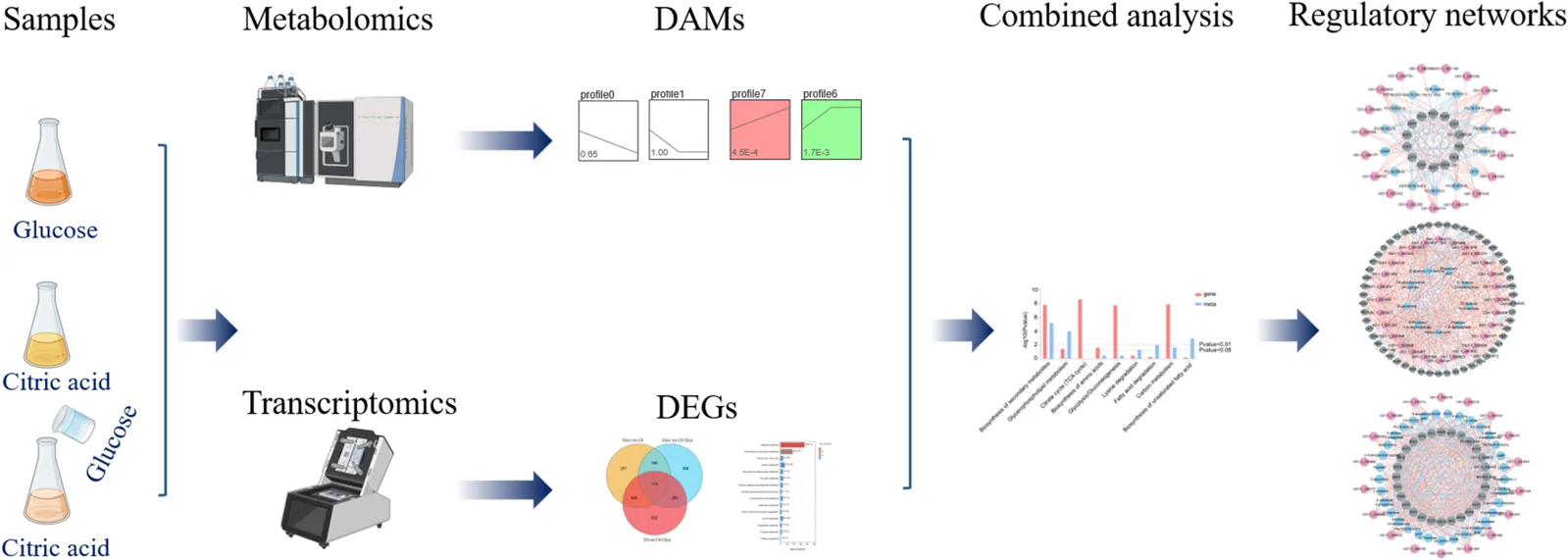

**Supplementary Information:**

The online version contains supplementary material available at 10.1007/s00253-025-13590-3.

## Introduction

The primary source of wine's acidity is derived from organic acids present in grapes and the metabolic activities of yeast strains, both of which significantly influence the quality of the wine. Challenges associated with alterations in the organoleptic properties of fruit wine have promoted intensive research on yeast-driven deacidification across different countries (Cimini and Moresi 2022; Del Fresno et al. 2017). In recent years, *Pichia* and *Issatchenkia* have stood out among the non-*Saccharomyces* genera due to their metabolic and enzymatic potential for bio-deacidification and their ability to generate distinctive characteristics (Barkhuizen et al. 2021; Cioch-Skoneczny et al. 2020; Drumonde-Neves et al. 2021; Du et al. 2019); nevertheless, the *Pichia kudriavzevii* species remains relatively under-researched.

*P. kudriavzevii* is a promising starter culture in the winemaking industry due to its ability to induce bio-deacidification, including the degradation of malic acid (del Mónaco et al. 2014; Kim et al. 2008), lactic acid (Kahve 2023) and citric acid (Zhong et al. 2021), and increase the overall aroma content (Holt et al. 2018; Liu et al. 2023; Zhang et al. 2021). At the same time, it also decreases the final ethanol levels (Rossouw and Bauer 2016) and the concentrations of ethyl carbamate that affect wine quality (Du et al. 2017). As *P. kudriavzevii* is a Crabtree-negative yeast species (Schnierda et al. 2014), it can grow with TCA (tricarboxylic acid cycle, TCA cycle) intermediates, such as citric, succinic, and malic acids, as sole carbon sources.

Nevertheless, the utilization of these acids by *P. kudriavzevii* is repressed by glucose in mixed carbon sources. *P. kudriavzevii* regulates carbon metabolism via carbon catabolite repression (CCR), preferentially consuming favorable substrates such as glucose while repressing the expression of genes involved in the utilization of non-preferred carbon sources (Liu et al. 2020; Singh et al. 2008). This regulatory mechanism facilitates rapid adaptation to environmental changes (Reuß et al. 2018). The inability or inefficient utilization of citric acid in the presence of glucose is a significant limitation that hinders the broader application of *P. kudriavzevii* for acid biodegradation or other chemical production processes. Relevant studies have verified the degradation of dicarboxylic acids in *P. kudriavzevii* (Kahve 2023; Zhu et al. 2020), but the precise signalling pathway of citric acid catabolism in response to glucose remains unclear (Xi et al. 2021). Therefore, further research is needed on the regulatory mechanism by which organic acid metabolism is inhibited by sugar in the fermentation matrix.

The glucose sensing and signalling pathway has been thoroughly investigated and characterized in *Saccharomyces cerevisiae* (Brink et al. 2021; Kalender and Çalık 2020) and filamentous fungi, such as *Aspergillus* sp (Reijngoud et al. 2021; Ries et al. 2021; Wang et al. 2024; Zheng et al. 2023). In yeast, major sugar signalling networks have been identified, including the Snf3/Rgt2, Snf1/Mig1 and the cyclic adenosine monophosphate (cAMP)/protein kinase A (PKA) pathway. Numerous studies have demonstrated that release of CCR can increase xylose utilization in the presence of glucose and reduce acetic acid level (Diaz et al. 2019; Shibata et al. 2021; Zhang et al. 2023). Therefore, investigating the mechanism of CCR in *P. kudriavzevii* could help identify gene-editing targets to improve the citrate utilization efficiency. However, the mechanism by which CCR inhibits citric acid-related catabolism and metabolism signalling networks in *P. kudriavzevii* remain largely unexplored (de Assis et al. 2018, 2021).

The practical application of *P. kudriavzevii* in biotechnology has lagged behind that of *S. cerevisiae* due to limited genetic information. However, this challenge has been alleviated by the availability of high-quality genomes (Cao et al. 2020; Tran et al. 2019). Omics techniques have been extensively employed to investigate the stress tolerance mechanisms of *P. kudriavzevii*, providing valuable genetic data for researchers to engineer strains with enhanced traits (Ruyters et al. 2014; Wang et al. 2020). Simultaneously, the knowledge gaps regarding the physiological mechanisms of CCR can be filled through omics techniques, thereby overcoming the limitations of industrial fermentation. The carbon repression mechanisms of *Lactobacillus plantarum* (Lu et al. 2018), *Candida glabrata* (Cottier et al. 2017), *Schizosaccharomyces pombe* (Sohn et al. 2012), *Yarrowia lipolytica* (Domenzain et al. 2022; Kavšček et al. 2015), and *Aspergillus nidulans* (de Assis et al. 2018; Zheng et al. 2023) were revealed via multi-omics analysis. More recently, the mining of multiomics information offers a template for exploring the CCR regulatory mechanisms of *P. kudriavzevii* at the molecular level.

We previously isolated the citrate-deacidification strain *P. kudriavzevii* GS1-1 (Wang and Liu 2013), which was found to contain CCR regulatory elements, including the *SNF1* complex and *MIG1* transcription factor-encoding genes. Here, to investigate whether the regulation of citric acid metabolism in GS1-1 also follows the classic CCR regulatory model, which involves the regulation of alternate carbon source utilization in response to glucose levels, multi-omics techniques involving RNA-seq (transcriptome sequencing) combined with meta-seq (metabolomic sequencing) were employed to analyze the expression and regulation of CCR-related genes and their influence on citrate acid-related metabolites.

Overall, the results obtained for GS1-1 revealed how citric acid utilization was affected by glucose-mediated CCR and revealed a global regulatory network of CCR. Our findings indicate that MIG1 and GCN4 might be important regulators of diverse processes in addition to carbon metabolism by modulating the hierarchy of different regulatory networks.

## Materials and methods

### Strain culture and sample preparation

The *P. kudriavzevii* GS1-1 strain, previously isolated from the Ningxia wine region of China (Wang and Liu 2013), was cultured at 30 °C for 48 h for purification. A single colony was inoculated in YPD medium, consisting of 1% yeast extract, 2% peptone, and 2% glucose, and incubated at 30 °C, 150 rpm for 17 h to prepare a yeast suspension. After microscopic counting, the suspension (1 × 10⁶ cells/mL) was introduced into 250 mL flasks containing 50 mL of sterile YPD and incubated at 30 °C, 150 rpm for 7 h. After centrifugation, the cell pellet was recollected and labeled as Gluc. Similarly, yeast cultivated in YPC medium (glucose replaced by 0.5% citric acid) for 17 h was designated as Cit. In another set of YPC medium, 2% glucose was added after 17 h of incubation, followed by an additional 1.5 h of incubation and centrifugation to yield the Cit-Gluc samples. All samples were stored at −80 °C prior to omics analysis. All strains used in this study have been deposited at the College of Enology, Northwest A&F University, Yangling, Shaanxi province, China, and are available from the corresponding author upon reasonable request for research and reproducibility purposes.

### Metabolite extraction and LC–MS/MS analysis

Cells (~ 10⁷ per sample) were washed with PBS at 37 °C, resuspended in 800 μL methanol/acetonitrile (1:1, v/v), sonicated for 30 min, and stored at −20 °C for 10 min. The supernatant was obtained via centrifugation (14,000 × g, 20 min) and analyzed using UPLC-MS/MS (Agilent 1290 Infinity LC system, Agilent Technologies, Santa Clara, CA, USA; AB Triple TOF 6600 mass spectrometer, AB Sciex, Concord, ON, Canada; Waters ACQUITY UPLC BEH Amide column, Waters Corporation, Milford, MA, USA). Quality control (QC) samples were prepared by mixing equal volumes of all test samples to monitor the reproducibility and stability of the analytical process. The chromatographic conditions included a mobile phase of water (25 mM ammonium acetate, 25 mM ammonia) and acetonitrile, a column temperature of 25 °C, a flow rate of 0.5 mL/min, and an injection volume of 2 μL. Mass spectrometry was performed under electrospray ionization (ESI) conditions, scanning in the 60–1000 Da m/z range. Data processing was conducted using the R package ropls (Thévenot et al. 2015). Differentially abundant metabolites were identified based on a variable importance in projection (VIP) score ≥ 1 and *P* < 0.05, as determined by orthogonal partial least squares discriminant analysis (OPLS-DA) and Student’s *t*-test (Saccenti et al. 2013). Metabolic pathway enrichment analysis was conducted using the Kyoto Encyclopedia of Genes and Genomes (KEGG) database.

### RNA sequencing and transcriptomics analysis

Total RNA was extracted from three biological replicates using an RNA isolation kit (Thermo Fisher Scientific, Waltham, MA, USA). Raw reads obtained via the Illumina HiSeq platform were filtered using fastp (v0.19.3) and mapped to the reference genome using HISAT2 (v2.1.0) (Kim et al. 2015). Gene expression was quantified as fragments per kilobase of exon model per million mapped fragments (FPKM) using featureCounts (v1.6.2; R package, Bioconductor). Differentially expressed genes (DEGs) were identified using DESeq2 (v1.22.1; R package, Bioconductor) with log₂|fold change|≥ 1 and false discovery rate (FDR) < 0.05 (Love et al. 2014). Pearson correlation analysis was performed using R (v4.0.3; R Foundation for Statistical Computing, Vienna, Austria), and DEG enrichment analysis was conducted via a hypergeometric distribution test.

### Gene network analysis and visualization

Transcription factor (TF) annotation was based on the JASPAR database (JASPAR 2022). Regulatory networks were constructed in three steps: (1) identifying structural genes involved in metabolic pathways through integrated metabolomic and transcriptomic analysis of Cit vs. Cit-Gluc; (2) correlating TFs with structural genes via Pearson correlation analysis; and (3) filtering differentially accumulated metabolites (DAMs) involved in glycerophospholipid, carbon, and secondary metabolism. Networks were visualized using Cytoscape (v3.8.0; Cytoscape Consortium, San Diego, CA, USA).

### RT-qPCR validation

Total RNA isolation and cDNA synthesis followed Wang et al. (2020). Expression levels of six DEGs (*SNF1, MIG1, PFK1, FBP1, RGT1, ACL*) were validated via RT-qPCR, with *ACT1* as an internal control. Relative expression was calculated using the 2^−ΔΔCt^ method (Adnan et al. 2011).

### Statistical analysis

Experiments followed a completely randomized design. Data were presented as mean ± standard error from at least three independent replicates. Statistical significance was assessed using Student’s *t*-test at *P* < 0.05 or 0.01.

## Results

### Metabolomic profiles of *P. kudriavzevii* in response to glucose repression

**Fig. 1:**
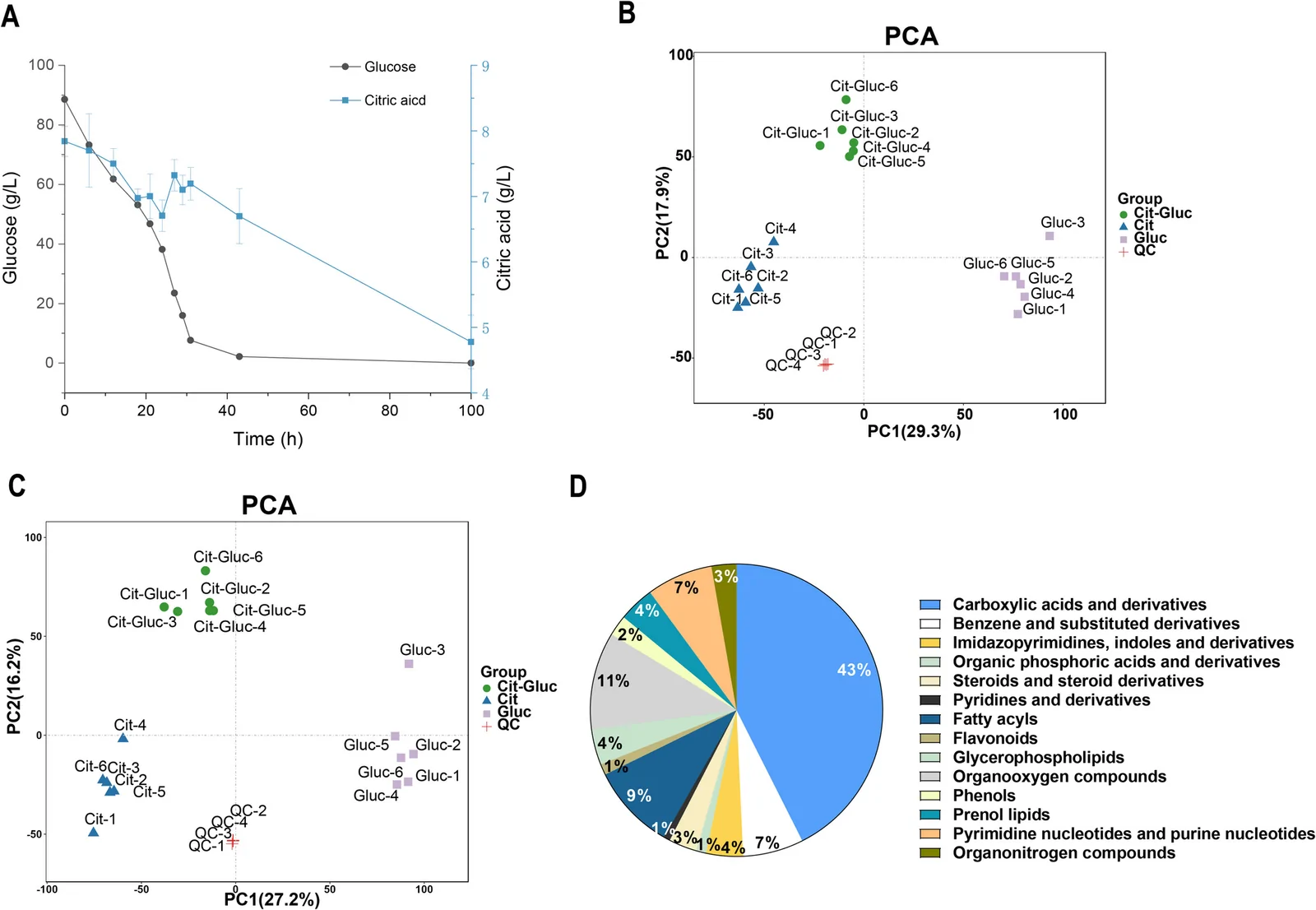
(**A**) Carbon utilization of *P. kudriavzevii* GS1-1 in dual carbon sources. PCA analysis of metabolomics data in (**B**) negative ion modern and (**C**) positive ion modern for samples fermented in three carbon sources. (**D**) Classification and proportion of identified metabolites

The changes in the carbon source during fermentation showed that glucose inhibited the utilization of citric acid, which could not conduct until the concentration of glucose was less than 2 g/L (Fig. 1A). Interestingly, citric acid concentration exhibited a non-linear pattern: it slightly decreased from 0 to 25 h, likely due to initial transportation, then transiently increased around 30 h, possibly reflecting overflow metabolism, before declining again as glucose was depleted and citrate catabolism activated. To better explore the metabolic dynamics of *P. kudriavzevii* strain GS1-1 in response to CCR, the changes in metabolites were monitored via an LC–MS/MS-based metabolic profiling method. Principal component analysis (PCA) revealed that the Cit, Gluc and Cit-Gluc samples were clearly separated by PC1 and PC2, accounting for 43.4% and 47.2% of the total variation in the positive and negative ion modes, respectively (Fig. 1B, C). From the PCA, it can be observed that the 18 samples were clearly divided into three groups. The intragroup samples (six biological replicates) showed significant correlations, which was consistent with the PCA results (Supplemental Fig. S1), indicating that the metabolomics data were highly replicable and qualified for further analyses. Further statistical analysis of the metabolites revealed that a total of 13,743 (positive ion mode) and 9,742 (negative ion mode) metabolites in GS1-1 during fermentation and substrate consumption; these metabolites were divided into 14 categories, including benzene and substituted derivatives, carboxylic acids and derivatives, fatty acyls, flavonoids, glycerophospholipids, imidazopyrimidines, indoles and derivatives, organic phosphoric acids and derivatives, organooxygen compounds, phenols, prenol lipids, pyridines and derivatives, steroids and steroid derivatives, pyrimidine nucleotides and purine nucleotides and organonitrogen compounds.

### DAMs of *P. kudriavzevii* GS1-1 in response to glucose repression

**Fig. 2:**
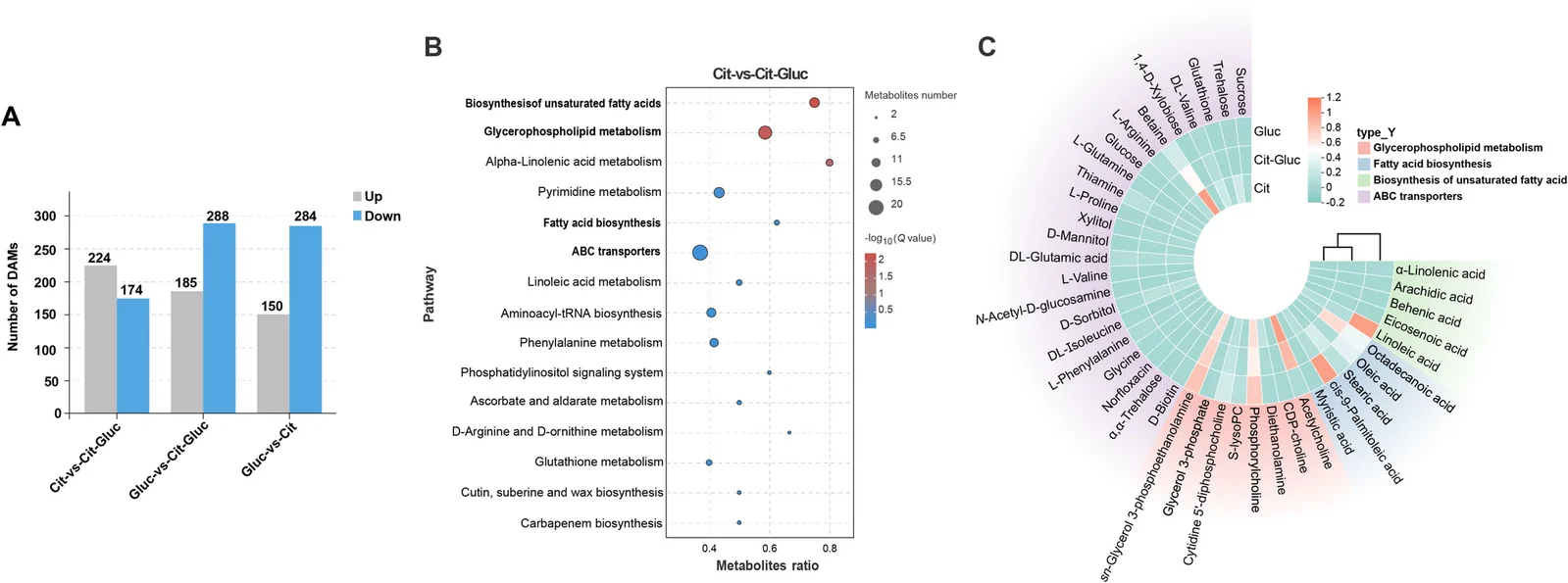
**A** The numbers of differentially up- and down-accumulated metabolites among samples fermented in three carbon source media by comparison of each group. **B** KEGG enrichment analysis of DAMs between Cit and Cit-Gluc. **C** The abundance of DAMs involved in the top enriched pathways across Cit, Gluc, and Cit-Gluc samples

Next, 670 differentially accumulated metabolites (DAMs) between the Cit and Cit-Gluc samples were identified using OPLS-DA to investigate the metabolic changes in GS1-1 in response to glucose addition (Supplemental Table S1, Supplemental Fig. S2 and S3). Metabolites with statistically significant changes (VIP ≥ 1 and absolute log_2_ |fold change|≥ 1) were screened to evaluate the DAMs. As depicted in Fig. 2A, a total of 398, 473, and 434 DAMs were detected between Cit and Cit-Gluc, between Gluc and Cit-Gluc, and between Gluc and Cit-Gluc, respectively, through pairwise comparisons. Overall, the number of DAMs varied with changes in carbon resources. Among these DAMs, there were 224 upregulated and 174 downregulated metabolites, 185 upregulated and 288 downregulated metabolites, and 150 upregulated and 284 downregulated metabolites in Cit compared with Cit-Gluc, Gluc compared with Cit-Gluc, and Gluc compared with Cit, respectively (Fig. 2A).

The top enriched KEGG terms between Cit and Cit-Gluc for all the identified DAMs were pathways related to the biosynthesis of unsaturated fatty acids, glycerophospholipid metabolism, and alpha-linolenic acid metabolism (Fig. 2B; Supplemental Table S2). The biosynthesis of unsaturated fatty acids, fatty acid biosynthesis, glycerophospholipid metabolism and ABC transporters were the common top enriched KEGG pathways of the DAMs in the three groups that were compared (Fig. 2B; Supplemental Table S2, Supplemental Fig. S4). For the biosynthesis of unsaturated fatty acids, 12, 8 and 9 DAMs with high VIP values were identified in the Cit-vs. Cit-Gluc, Gluc-vs.-Cit-Gluc, and Gluc-vs.-Cit comparisons, respectively (Supplemental Table S2). The levels of most DAMs in the unsaturated fatty acid, fatty acid biosynthesis and glycerophospholipid metabolism pathways were greater in the Cit-Gluc samples than in the Cit samples (Fig. 2B; Supplemental Table S3). However, in ABC transporters, the abundances of carboxylic acids and derivatives and organooxygen compounds, including L-glutamine, betaine, trehalose, L-proline, L-arginine, and DL-valine, decreased when glucose was added, implying that their transport was repressed by glucose. These results suggest that glucose enhances the accumulation of certain fatty acids, unsaturated fatty acids and glycerophospholipids; thus, glucose represses the transport of carboxylic acids and derivatives and organooxygen compounds and possibly inhibits citric acid catabolism.

**Fig. 3:**
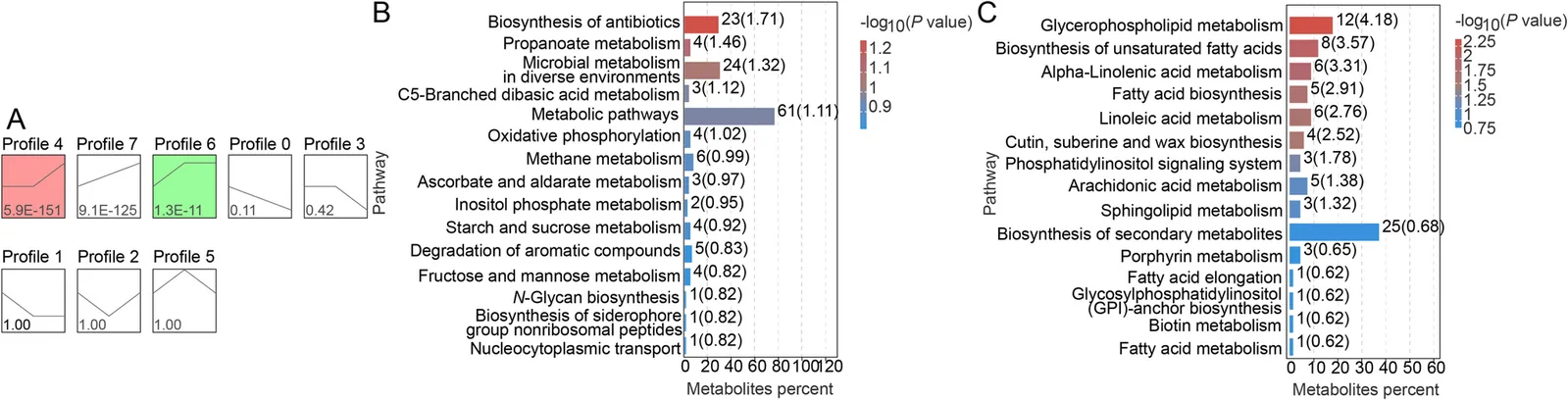
**A** Trend analysis plot of DAMs in response to different carbon sources (Cit vs. Cit-Gluc vs. Gluc), showing designated profiles 1 through 7. **B** KEGG pathway enrichment analysis of DAMs in the selected profile 0 and profile 1. **C** KEGG pathway enrichment analysis of DAMs in the selected profile 6 and profile 7

To identify crucial DAMs, a trend analysis was conducted (Cit-vs.-Cit-Gluc-vs.-Gluc). DAMs were classified into seven distinct clusters (profiles 1–7) according to their accumulation patterns (Fig. 3A), suggesting that the identified metabolites accumulated or decreased diversely under various carbon conditions. Specifically, profile 6 and profile 7 reflected high accumulation of metabolites, while glucose delayed the decline of these metabolites; profile 0 and profile 1 reflected high degradation of metabolites, and glucose retarded the accumulation of these metabolites.

Further investigation of the functions of the grouped metabolites revealed that the significantly enriched pathways in profiles 6 and 7 were glycerophospholipid metabolism, unsaturated fatty acid biosynthesis, alpha-linolenic acid metabolism, fatty acid biosynthesis, linolenic acid metabolism, cutin, suberine and wax biosynthesis, and the phosphatidylinositol signalling system. Based on the significantly enriched pathways of profile 0 and profile 1, the accumulation of metabolites involved in the biosynthesis of antibiotics, propanoate metabolism, microbial metabolism in diverse environments, C5-branched dibasic acid metabolism, and metabolic pathways in diverse environments were repressed by glucose (Fig. 3B, C; Supplemental Table S4). Hence, these results suggest that these metabolic and biosynthetic pathways may lead to changes in biochemical compounds involved in glucose-retarded citrate catabolism and help maintain the sequences of carbon sources utilized.

### Transcriptomic analysis of glucose repression

**Fig. 4:**
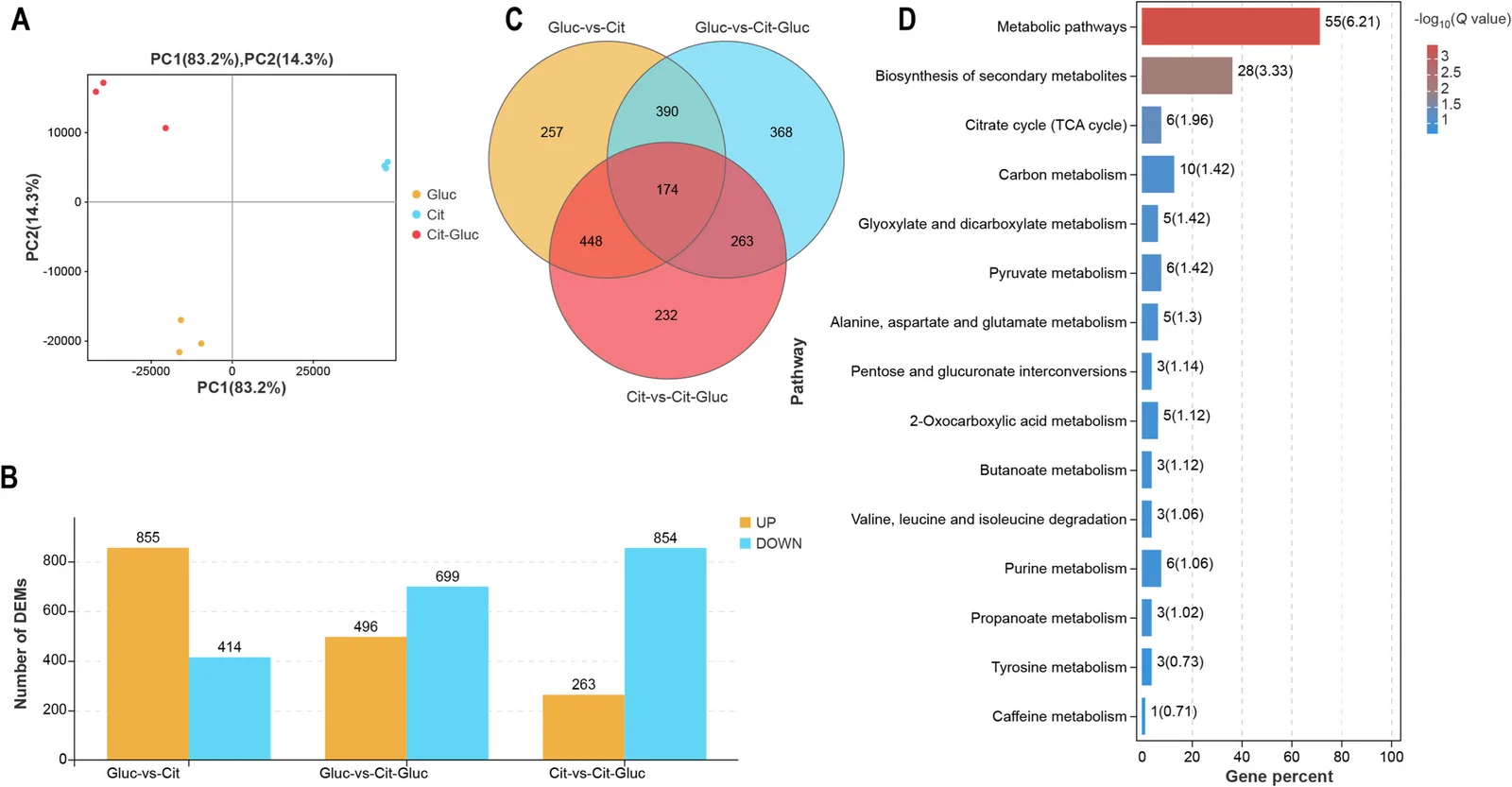
Transcriptome analysis of *P. kudriavzevii* GS1-1 cultured in three carbon resources. **A** PCA of the nine samples for transcriptomics analysis. **B** The number of up-regulated and down-regulated DEGs between diverse carbon condition in each comparison group respectively. **C** Venn analysis of 2,132 DEGs. (D) KEGG enrichment analysis of DEGs in the most overlapping area

High-throughput RNA-seq was then conducted to further investigate the transcriptional modulatory mechanisms by which glucose inhibits citrate catabolism and maintains carbon utilization. Total clean data ranging from 20,153,236 to 30,028,434 for each library remaining (99.35% to 99.51%) were generated after low-quality reads and adaptor sequences were eliminated. The Q30 percentage was greater than 92%, and the GC content was greater than 42%. Approximately 97.37% to 99.38% of the clean reads from each library were mapped to the reference genome (Supplemental Table S5). Moreover, PCA illustrated that all the biological replicates were clustered together, revealing substantial differences among the samples fermented with diverse carbon sources (Fig. 4A). As outlined above, these results demonstrated a highly reliable bioinformatics analysis of our RNA-seq data for diverse carbon source-fermented GS1-1.

### DEGs involved in glucose repression

According to the thresholds of log_2_ |fold change|≥ 1 and FDR < 0.05, there were 1117, 1195, and 1269 DEGs in the comparisons of Cit and Cit-Gluc, Gluc and Cit-Gluc, and Gluc and Cit, respectively (Fig. 4B). By Venn diagram analysis, all DEGs were divided into seven groups according to their overlapping areas (Fig. 4C). By investigating the potential functions of 174 DEGs in the most overlapping area, we found that the expression of genes related to citrate utilization was inhibited by glucose. These genes were mainly involved in metabolic pathways, biosynthesis of secondary metabolites, citrate circle, carbon metabolism, glyoxylate and dicarboxylate metabolism, pyruvate metabolism, alanine, aspartate and glutamate metabolism, pentose and glucuronate interconversions, and 2-oxocarboxylic acid metabolism (Fig. 4D). These results implied that these metabolic pathways are regulatory pathways involved in glucose-maintained carbon utilization during citrate catabolism.

### Combined analysis of metabolomics and transcriptomics

**Fig. 5:**
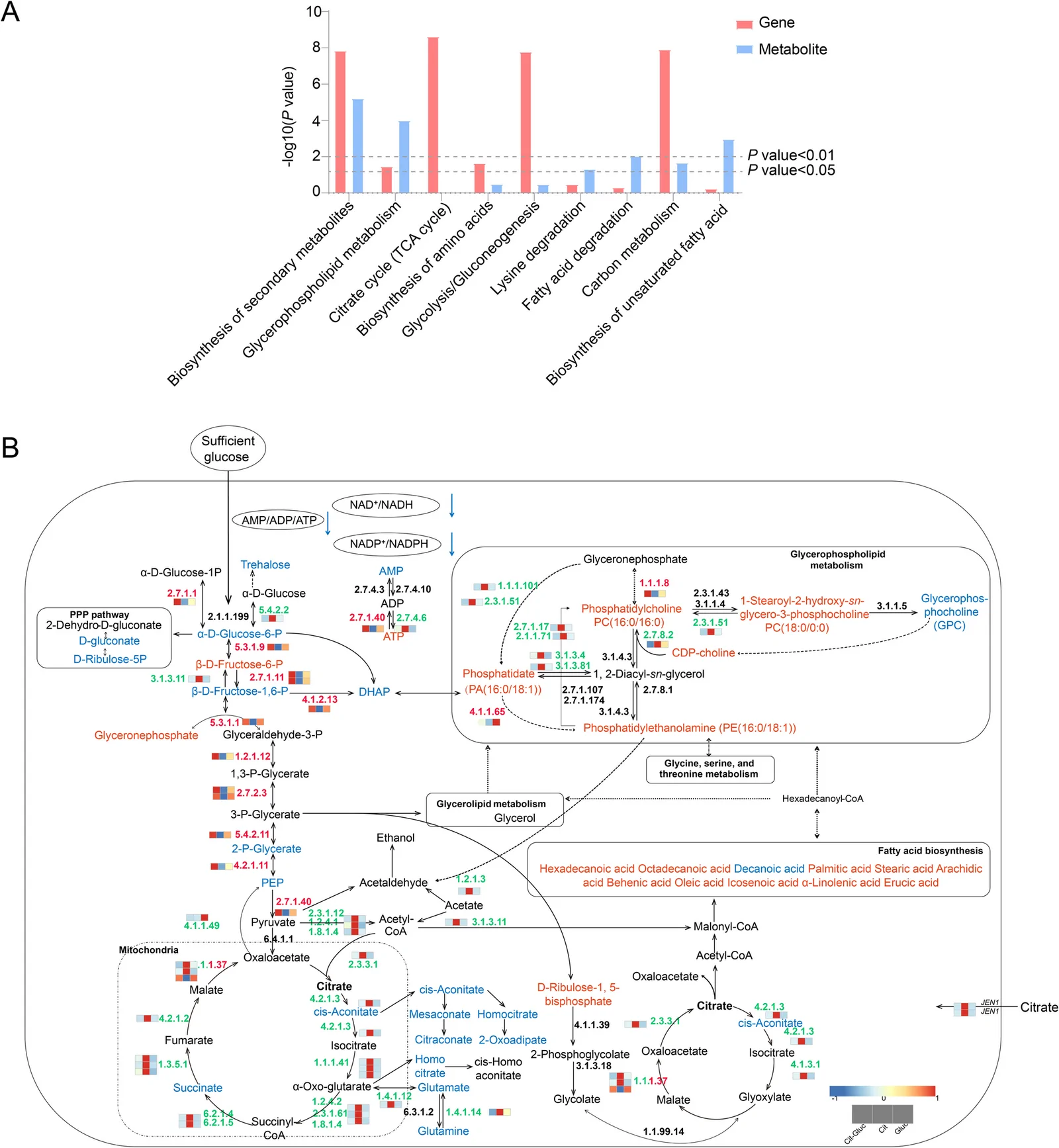
Integrated analysis of transcriptomic and metabolomic data. **A** Joint KEGG enrichment *P*-value histogram. **B** Pathway analysis of DEGs and DAMs associated with glycerophospholipid metabolism, carbon metabolism and biosynthesis of secondary metabolites. EC numbers marked in red and green indicate higher or lower levels of DEGs, respectively, in Cit-Gluc samples relative to Cit samples. Metabolites marked in red and blue indicate higher or lower levels of DAMs, respectively, in Cit-Gluc samples relative to Cit samples

A joint KEGG pathway analysis of DEGs and DAMs was conducted to further identify metabolic pathways associated with the inhibition of citrate catabolism. The significantly enriched pathways (*P* < 0.01) included carbon metabolism, biosynthesis of secondary metabolites and glycerophospholipid metabolism, which are associated with citric acid catabolism and CCR (Fig. 5A). DEGs and DAMs that mapped simultaneously to related pathways were illustrated in a pathway map to clarify the role of these metabolic pathways in the inhibition of glucose (Fig. 5B). By integrating metabolomics and transcriptomics data, we determined that the differentially expressed genes are closely correlated with variations in metabolites in GS1-1 during citrate catabolism. These results revealed that gene expression patterns are associated with metabolic pathways involved in the repression of deacidification by glucose.

The contents of most glycerophospholipid compounds in GS1-1 tended to increase in response to glucose addition (Fig. 2C; Supplemental Table S3). As shown above, the accumulation of dihydroxyacetone phosphate (DHAP) and glycerophosphocholine (GPC) was decreased by glucose in GS1-1 (Supplemental Table S6). We next attempted to identify the regulators that control the glycerophospholipid metabolism pathway. Based on our clustering data and KEGG analyses, 15 DEGs were enriched in the glycerophospholipid metabolism pathway, and the expression of 12 DEGs was greater in Cit-Gluc samples than in Cit samples. Among these DEGs, the expression of DEGs encoding phosphatidylethanolamine (*PEMT*, EC 2.1.1.17 2.1.1.71), phosphatidate phosphatase (*DPP1*, EC 3.1.3.4 3.1.3.81), 1-acylglycerone phosphate reductase (*AYR1*, EC 1.1.1.101), lysophospholipid acyltransferase (*ALE1*, EC 2.3.1.51) and diacylglycerol cholinephosphotransferase (*CPT1*, EC 2.7.8.2) was greater in Cit samples than in Cit-Gluc samples (Fig. 5B). The expression of the DEGs encoding glycerol-3-phosphate dehydrogenase (*GPD1*, EC 1.1.1.8) and phosphatidylserine decarboxylase (*PISD*, EC 4.1.1.65) was lower in the Cit samples than in the Cit-Gluc and Gluc samples (Fig. 5B). The results showed that *GPD1* and *PISD* negatively regulated the degradation of DHAP and GPC after glucose was added (Fig. 5B; Supplemental Table S6). In the carbon metabolism pathway, 54 critical DEGs related to carbon metabolism and organic acid catabolism were identified. Notably, the expression levels of genes encoding fructose-1,6-bisphosphatase I (*FBP1*, EC 3.1.3.11) and phosphoenolpyruvate carboxykinase (ATP) (*PCKA*, EC 4.1.1.49), both involved in gluconeogenesis, were significantly downregulated upon glucose addition. In the TCA cycle and glyoxylate cycle, the expression of the malate dehydrogenase-encoding gene (*MDH2*, EC 1.1.1.37) was greater in Cit-Gluc samples than in Cit samples (Fig. 5B; Supplemental Table S6). After glucose was added, most of the DEGs related to gluconeogenesis, the TCA cycle and the glyoxylate cycle were gradually downregulated, whereas the DEGs related to glycolysis were highly upregulated. In addition, by targeting the biosynthesis of secondary metabolite pathways among the DEGs, we identified 28 structural genes (Supplemental Table S6).

### Construction of regulatory networks based on key transcription factors

Transcription factors (TFs) are essential for regulating carbon source utilization in yeast strains. Based on analysis of the structures of the transcripts in the candidate DEG set via Venn analysis, we detected 81 TFs belonging to 10 TF families, which mainly consisted of 5 bHLH TFs, 5 bZIP TFs, 29 C_2_H_2_ TFs, 7 C_3_H, 1 FAR1 TF, 6 GATA TFs, 3 HB-other TFs, 3 HSF TFs, 2 M-type TFs, 11 MYB TFs, and 9 NF TFs (Supplemental Table S7). We then analysed the expression levels of these TF genes. The heatmap showed that the expression of most of the candidate TFs significantly decreased with the addition of glucose (Supplemental Fig. S5).

**Fig. 6:**
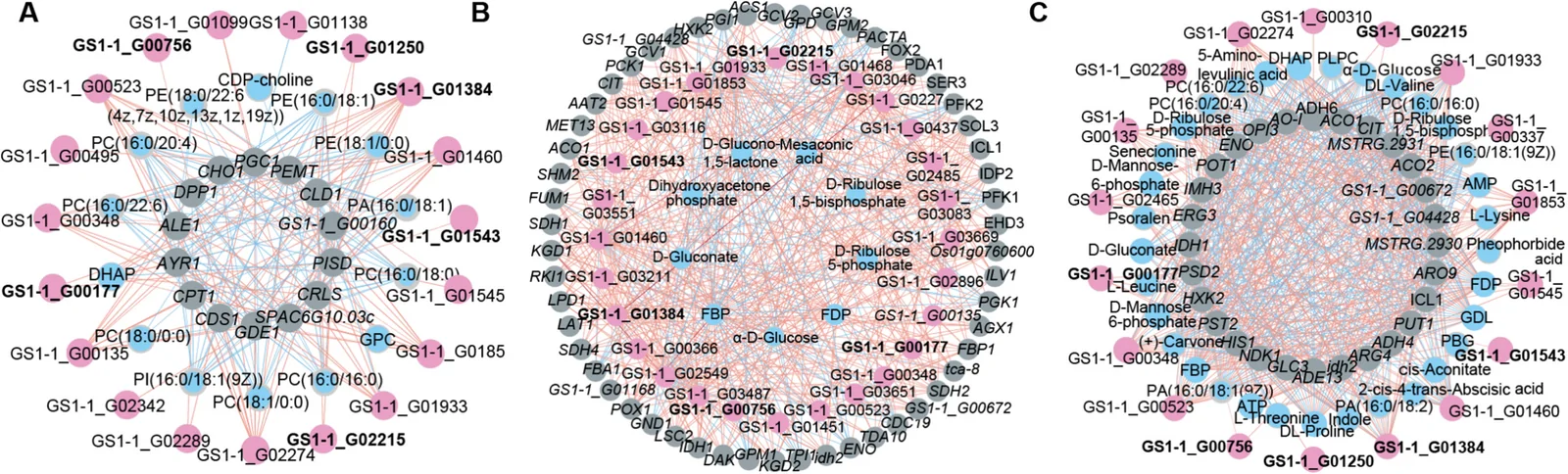
The putative transcriptional metabolic regulatory network of key metabolism in CCR-related acid catabolism. **A** Glycerophospholipid metabolism pathway. **B** Carbon metabolism pathway. **C** Biosynthesis of secondary metabolites. Gray, pink and blue circles represent the structural genes of the corresponding pathways, different families of TFs identified whose transcripts are correlated with expression of structural genes, and the differentially expressed metabolites of the relevant pathways whose values are associated with the expression of structural genes, respectively. Transcription factors (TFs) commonly associated with the three pathways are highlighted in bold

Furthermore, by correlating the transcript accumulation of DEGs associated with glycerophospholipid metabolism, carbon metabolism and biosynthesis of secondary metabolites, a total of 19, 28 and 18 candidate TFs were found based on Pearson’s correlation coefficient among the DEGs, respectively (Supplemental Table S7). The potential regulatory networks mediated by these TFs were then constructed and visualized (Fig. 6). All these TFs were strongly correlated with the DEGs involved in each metabolic pathway of CCR (Fig. 6; Supplemental Table S8).

Pearson correlation analysis between DEGs and DAMs associated with glycerophospholipid, carbon and secondary metabolites during glucose repression was performed to integrate the metabolomics and transcriptomics data (Fig. 6). Based on Pearson Correlation Coefficient (PCC), the results showed that changes in the relative contents of 14 glycerophospholipid metabolites (|PCC|> 0.80) belonged to profiles 0, 1, 6 and 7, contrary to glycerophospholipid structural gene expression. These metabolites included GPC, DHAP, CDP-choline (cytidine diphosphate choline), PC (phosphatidylcholine) (18:1/0:0, 16:0/16:0, 18:0/0:0, 16:0/22:6, 16:0/18:0, 16:0/20:4), PI (phosphatidylinositol) (16:0–18:1(9z)), PE (phosphatidylethanolamine) (18:0/22:6(4z,7z,10z,13z,16z,19z), 16:0/18:1, 18:1/0:0), and PA (phosphatidic acid) (16:0/18:1). These results suggested that these TF families participate in upregulating potential glycerophospholipid metabolites by repressing the expression of these structural genes in response to glucose treatment (Fig. 6A; Supplemental Table S6). With a combination of transcriptomics and metabolomics, a total of 9 and 33 metabolites were strongly co-expressed with the DEGs in carbon metabolism pathway (|PCC|> 0.95) and biosynthesis of secondary metabolites (|PCC|> 0.90), respectively (Fig. 6B and C; Supplemental Table S7 and S8). In the TCA cycle and glyoxylate cycle, the accumulation of succinate and cis-aconitate was repressed when glucose was added. Furthermore, the accumulation of homocitrate, citraconate glutamate and glutamine, which are downstream metabolites of citrate, was prevented by glucose. In the glycolysis/gluconeogenesis pathway, the levels of trehalose, α-D-glucose-6-P, β-D-fructose-1,6-P, 2-P-glycerate and phosphoenolpyruvate (PEP) were lower in Cit-Gluc samples than in Cit samples, whereas the level of β-D-fructose-6-P was greater. ATP and D-ribulose 1,5-bisphosphate were the secondary metabolites that were more abundant in the Cit-Gluc samples than in the Cit samples (Fig. 5B). The results suggested that these metabolites perform important regulatory functions that inhibit citrate catabolism in the presence of glucose.

### RT-qPCR validation of RNA-seq data

**Fig. 7:**
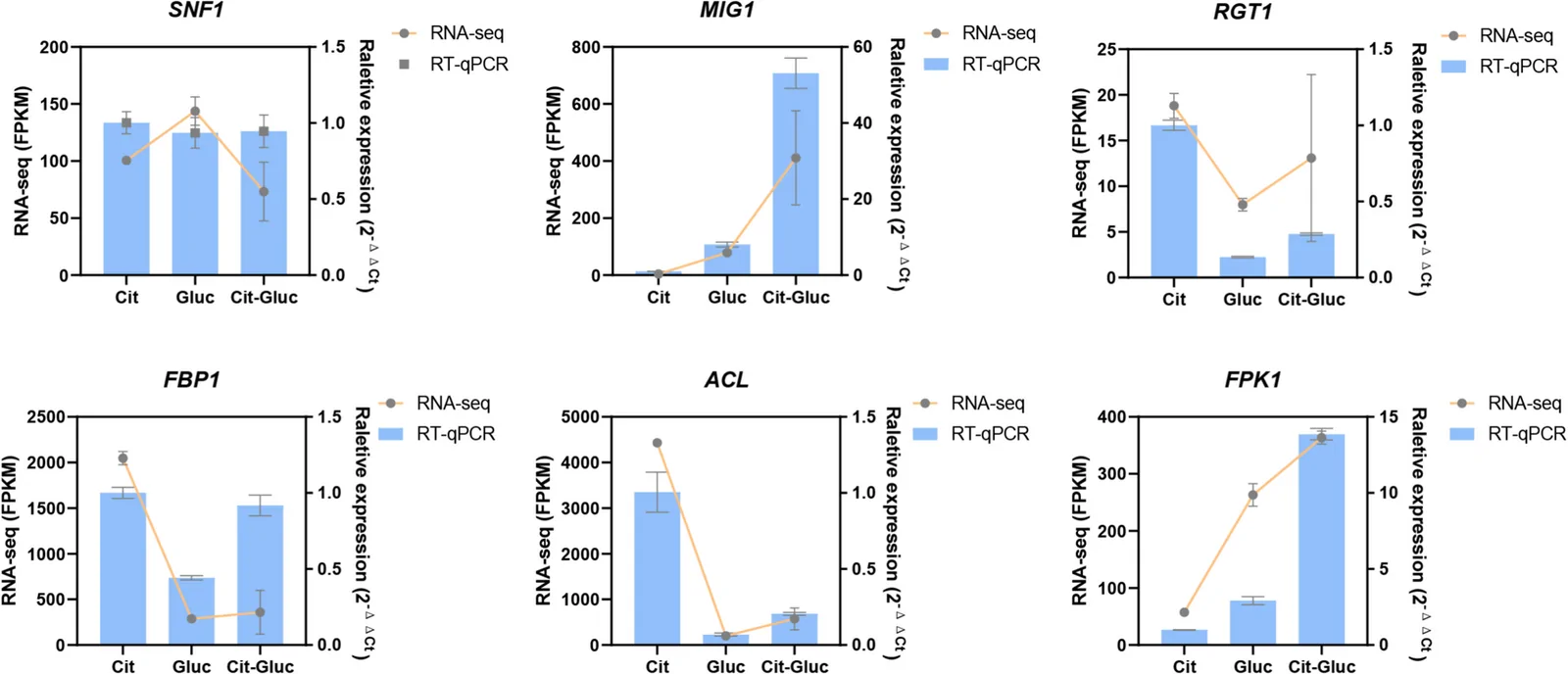
Validation of RNA-seq data by RT-qPCR analysis. Expression pattern validation of 6 selected DEGs in the RNA-seq analysis by RT-qPCR in Cit, Gluc and Cit-Gluc. The histograms were plotted using data obtained by RT-qPCR and the corresponded line chart was plotted by FPKM values in the RNA-seq analysis

To validate the accuracy of our RNA-seq data and explore the expression of key genes involved in citrate metabolism, six DEGs (*SNF1*, *MIG1*, *PFK1*, *FBP1*, *RGT1* and *ACL*) were selected based on their known roles in glucose repression and central carbon metabolism, as supported by both our data and previous studies. RT-qPCR analysis revealed expression patterns that were largely consistent with the RNA-seq results (Fig. 7), confirming the reliability of the transcriptome data.

## Discussion

### Carbon source shifts reprogram central metabolism

The acid-tolerant yeast *P. kudriavzevii* GS1-1 exhibits broad metabolic flexibility, utilizing both common carbon sources and Krebs cycle intermediates (Suthers et al. 2020). Like many microorganisms, it undergoes catabolite repression, where glucose suppresses genes involved in the utilization of alternative substrates such as citric acid (Zhong et al. 2021). The results of the combined transcriptomic and metabolomic analyses revealed that glycerophospholipid metabolism, carbon metabolism, and secondary metabolite biosynthesis are key pathways affected during CCR (Fig. 5A). The RT-qPCR validation of six representative genes supported the RNA-seq data and revealed carbon source-dependent regulatory patterns (Fig. 7). Upregulation of *MIG1* and *PFK1* under Cit-Gluc indicated glucose-mediated repression of citrate metabolism and activation of glycolysis. Conversely, downregulation of *FBP1* and *ACL* reflected suppressed gluconeogenesis and citrate-derived acetyl-CoA production. The expression trends of *SNF1* and *RGT1* further supported CCR involvement. These results underscore coordinated transcriptional regulation and metabolism reprogram in response to carbon source shifts. Given the time lag between transcript expression and protein accumulation (Balcerowicz et al. 2021), transcriptomic data provide insights into early events in CCR regulation.

### Membrane remodeling via glycerophospholipid reprogramming

Glycerophospholipid metabolism is crucial for maintaining membrane integrity under stress conditions, including phosphatidylcholine (PC)-induced stress (Tan et al. 2021). Differentially expressed metabolites such as PC (16:0/16:0), phosphatidylserine, and phosphatidic acid have been identified under stress conditions. In *S. cerevisiae*, a 30% decrease in phosphatidic acid was observed under cinnamic acid stress (Guo et al. 2018). In this study, an 85% decrease in glycerophosphocholine and an 857% increase in PC (16:0/16:0) were detected in *P. kudriavzevii* GS1-1 with glucose supplementation, indicating adjustments of glycerophospholipid to environmental glucose (Supplemental Table S6; Fig. 5B).

### Fatty acid metabolic shifts and regulatory mechanisms

Fatty acid metabolism plays a central role in membrane structure and energy production (Tojo et al. 2011). The degradation and biosynthesis of fatty acids must be finely regulated to maintain lipid homeostasis. ATP citrate lyase (ACL), which converts cytosolic citrate to acetyl-CoA, is essential for fatty acid synthesis (Fatland et al. 2005). In this study, the genes encoding acyl-CoA oxidase (*AOX1*, EC1.3.3.6) and *ACL* (EC 2.3.3.8) were significantly downregulated upon glucose addition (Fig. 5B). Fatty acids, particularly unsaturated types such as palmitoleic and oleic acids, are integral to yeast membranes (Li et al. 2021). Differential expression of unsaturated fatty acid metabolites was observed among samples with different carbon sources, including increased levels of alpha-linolenic, arachidic, behenic, eicosenoic, octadecanoic, oleic, and stearic acids in glucose-supplemented conditions (Fig. 2C; Supplemental Table S3). These fatty acids were slowly released as triglycerides via peroxisome β-oxidation in citric acid media (Tsigie et al. 2011). The findings suggested that glucose represses citric acid metabolism by inhibiting fatty acid-to-lipid conversion, as free fatty acid degradation ceases when cells transition to a nutrient-rich medium (Gonçalves et al. 2014).

### *MIG1*-linked regulatory network suppresses citrate utilization

Further, 19 glycerophospholipid-related transcription factors (TFs) were identified (|PCC|> 0.90). Genes encoding *MIG1* (GS1-1_G00177), *SPAC25B8.19c* (GS1-1_G00756), *JJJ1* (GS1-1_G01384/GS1-1_G01250), *YPR022C* (GS1-1_G01543), and *GCN4* (GS1-1_G02215) were upregulated in glucose conditions (Fig. 6A; Supplemental Fig. S5). Interestingly, catabolite control protein A (CcpA), a homolog of MIG1 in *Lactobacillus plantarum*, regulates the utilization of mixed carbon sources by directly binding to the promoter regions of target genes or indirectly influencing local regulators involved in fatty acid biosynthesis (Lu et al. 2018). In addition, CcpA exerts a dual role in fructooligosaccharide metabolism (FOS metabolism) via both direct and indirect regulatory mechanisms (Chen et al. 2019). The results suggested MIG1 may play a role in citric acid catabolism by directly repressing citric acid metabolism genes or by regulating glycerophospholipid- and fatty acid-related genes. However, the exact regulatory mechanism remains unclear.

### Metabolic pathway reprogramming in response to carbon sources

Carbon metabolism pathways determine cellular responses to glucose environments. Expression of the ribose 5-phosphate isomerase A-encoding gene (*RPIA*, EC 5.3.1.6) increased 7- to tenfold in samples using citric acid as the sole carbon source (Supplemental Table S6), suggesting ribosomal adaptation to environmental shifts (Sun et al. 2020). Genes encoding ATP phosphoribosyl transferase (*HIS1*, EC 2.4.2.17) increased by 327% under citric acid conditions, whereas mitochondrial malate dehydrogenase (*MDH2*, EC 1.1.1.37), isocitrate dehydrogenase (*IDH3*, EC 1.1.1.41) and fructose-1,6-bisphosphatase I (*FBP1*, EC 3.1.3.11) were significantly downregulated in Cit-Gluc (Supplemental Table S6; Figs. 5B, 6B). Trehalose accumulation, a gluconeogenesis end product, was repressed in glucose conditions, implying glucose suppresses citric acid utilization via gluconeogenesis and mitochondrial respiration repression (Giardina et al. 2012).

Additionally, genes encoding beta-oxidation proteins (*FOX2*, EC 4.2.1.- 1.1.1.-) and acyl-CoA oxidase (*AOX1*, EC 1.3.3.6) were downregulated with glucose supplementation (Supplemental Table S6; Figs. 5B, 6B), indicating inhibition of fatty acid and dicarboxylic acid catabolism (Beopoulos et al. 2009; Gonçalves et al. 2014). When glucose is unavailable, cells shift to beta-oxidation and alternative energy sources (Kloska et al. 2023). The glyoxylate cycle, producing succinic acid from fatty acid oxidation, is dependent on isocitrate lyase (ICL) and malate synthase. *ICL1* (EC 4.1.3.1) expression decreased by 87% upon glucose addition (Supplemental Table S6; Figs. 5B, 6B), corroborating findings in *S. cerevisiae* where glucose led to *ICL1* degradation (Sandai et al. 2012). In *Y. lipolytica*, beta-oxidation and lipid biosynthesis require ACL enzymes to generate acetyl-CoA (Friedlander et al. 2016). Thus, the results of the present study supported the notion that glucose inhibited the *ACL* (EC 2.3.3.8), preventing citric acid catabolism to downstream products, including fatty acids and lipids, and further repressing the glyoxylate cycle due to citric acid accumulation (Fig. 5B). Ribosomal responses to glucose include increased protein synthesis and nutrient absorption, while glycolysis/gluconeogenesis, mitochondrial function, and dicarboxylic acid metabolism are repressed.

### Secondary metabolism and energy homeostasis under CCR

Secondary metabolites, including carbohydrates, carboxylic acids, and amino acids, play a critical role in fermentation due to their energy transformation function. Carboxylic acids serve as metabolic intermediates under aerobic and anaerobic conditions. Snf1 kinase senses AMP/ADP/ATP levels and regulates CCR repression factors such as MIG1, affecting downstream gene expression (Shashkova et al. 2015). This study confirmed a decrease in the AMP/ATP ratio upon glucose supplementation (Figs. 5B, 6C), consistent with prior findings. Pyruvate kinase (*PK*, EC 2.7.1.40) was upregulated in glucose conditions, supporting the hypothesis that energy balance influences carbon metabolism (Fig. 5B; Supplemental Table S6).

### Integrated transcriptional control of metabolic networks

The synthesis of secondary metabolites in GS1-1 is controlled by structural genes and TFs (Fig. 6C). Transcriptomic analyses indicated that glucose significantly increased secondary metabolite biosynthesis gene expression, paralleling increased metabolite levels (Fig. 5B). These structural genes may play significant roles in the accumulation of certain carbohydrates, carboxylic acids and derivatives, amino acids and energy compounds. Five TFs—MIG1 (GS1-1_G00177), SPAC25B8.19c (GS1-1_G00756), JJJ1 (GS1-1_G01384/GS1-1_G01250), YPR022C (GS1-1_G01543) and GCN4 (GS1-1_G02215)—regulate both glycerophospholipid metabolism and secondary metabolite biosynthesis, and their expression was upregulated by glucose (Fig. 6C; Supplemental Fig. S5). This study observed increased expression of amino acid biosynthesis genes, including those for L-lysine, L-aspartic acid, L-leucine, L-threonine, and L-citrulline, in glucose conditions, alongside reduced levels of glutamate and glutamine, key amino acid precursors. Under low glucose conditions, GCN4, an activator protein in the amino acid biosynthesis pathway, was reported to be repressed by Snf1 kinase, downregulated amino acid biosynthesis (Shirra et al. 2008), and upregulated fatty acid synthesis, at least during glucose derepressing conditions (Woods et al. 1994). Moreover, the downregulation of glutamate and glutamine upon glucose addition was accompanied by reduced *MIG1* expression in citrate but significantly increased levels in glucose. Network analysis revealed strong connectivity between key TFs and metabolic pathways, suggesting their role in repressing citrate catabolism via glucose (Fig. 6; Supplemental Table S8). Additionally, *JEN1*, a citrate transporter, exhibited a 33-fold lower expression when glucose was present, supporting its inhibition of carboxylic acid transport (Xi et al. 2021).

Compared with prior research on carbon sources such as xylose, cellulose, and lactic acid in *Aspergillus*, this study uniquely explores citric acid metabolism in *P. kudriavzevii*. We found that 15, 54 and 28 DEGs involved in glycerophospholipid metabolism, carbon metabolism and secondary metabolite biosynthesis, respectively, were potentially modulated by 19, 28 and 18 TFs and 2 crucial TFs from the C_2_H_2_ family (Figs. 5A, 6). Findings indicate that glucose modulates carbon sensing and energy levels through MIG1 and GCN4, altering gene expression and metabolite accumulation in glycerophospholipid metabolism, carbon metabolism, and secondary metabolite biosynthesis. These findings advance the understanding of CCR regulation in non-*Saccharomyces* yeasts and highlight the need for further molecular investigations.

## Conclusions

The data in the present study showed that glucose strongly dampened citrate acid catabolism in *P. kudriavzevii* GS1-1. Metabolomics demonstrated that glucose repressed the β-oxidation of fatty acids and the accumulation of glycerophospholipids (GPC and DHAP) and maintained higher levels of sugar (trehalose, dihydroxyacetone phosphate, D-glucono-1,5-lactone, D-ribulose 5-phosphate, D-gluconate and D-fructose 1,6-bisphosphate) and amino acids (threonine, L-lysine, L-aspartic acid, L-leucine, L-threonine and L-citrulline) in the CCR. Furthermore, numerous secondary metabolites (ATP, AMP, gluconate and cis-aconitate) may play a role in the repression of citrate acid catabolism by glucose. Based on the integrated analysis of transcriptomics and metabolomics, three potential metabolic pathways (glycerophospholipid metabolism, carbon metabolism and biosynthesis of secondary metabolites) were inferred to be associated with CCR. In these metabolic pathways, the crucial TFs MIG1 and GCN4 and functional downstream genes, including *GPD1*, *PISD*, *FBP1*, *MDH*, *HIS1*, *RPIA*, *IDH3*, *ICL1*, *JEN1* and *ACL,* which might be related to the repression of citrate utilization, were successfully identified. In summary, our data provide a map of the metabolomes and transcriptomes of GS1-1 during the citric acid catabolism response to glucose, provide insight into the transcriptional control and regulatory networks of CCRs, and offer guidance for the future development of *P. kudriavzevii* as a promising starter culture. This study provides important information and new insight for the application of alleviating CCR mediated by glucose to enhance the deacidification capacity of *P. kudriavzevii* in wine fermentation.

## Supplementary Information

Below is the link to the electronic supplementary material.

ESM 1 (PDF 929 KB)

ESM 2 (XLSX 198 KB)

## Data Availability

Sequence data that support the findings of this study have been deposited in the National Center for Biotechnology Information with the primary accession code PRJNA1229810.
