# Supplementary material for: Multi-omics reveals glucose repression of citric acid catabolism in Pichia kudriavzevii
Source: Appl Microbiol Biotechnol. 2025 Sep 16;109(1):203. doi: 10.1007/s00253-025-13590-3 (PMC12441098; doi:10.1007/s00253-025-13590-3)
Supplement: Supplementary file 1 — (PDF 929 KB) [file 253_2025_13590_MOESM1_ESM.pdf]

Applied Microbiology and Biotechnology

Supplementary Material

Multi-omics reveals glucose repression of citric acid catabolism in *Pichia kudriavzevii*

Yichao Cheng[1], Xinyi Wang[1], Di Wu[1], Yao Lu[1], Yi Qin[1, 2], Yanlin Liu[1, 2], Yanying Liang[1] and Yuyang Song[1, 2*]

[1] College of Enology, Northwest A&F University, Yangling, Shaanxi 712100, China

[2] Ningxia Helan Mountain's East Foothill Wine Experiment and Demonstration Station of Northwest A&F University, Yongning, Ningxia 750104, China

[*]Address correspondence to Yuyang song: yuyangsong@nwsuaf.edu.cn

Supplementary information description

Figure captions

Figure S1. Pearson's correlation coefficients of different fermented samples in (A) negative ion mode and (B) positive ion mode.

Figure S2. The OPLS-DA scores of metabolites in negative ion mode between (A) Cit and Cit Gluc, (C) Gluc and Cit-Gluc and (E) Gluc and Cit; and the OPLS-DA loading of metabolites in negative ion mode between (B) Cit and Cit-Gluc, (D) Gluc and Cit-Gluc and (F) Gluc and Cit.

Figure S3. The OPLS-DA scores of metabolites in positive ion mode between (A) Cit and Cit Gluc, (C) Gluc and Cit-Gluc and (E) Gluc and Cit; and the OPLS-DA loading of metabolites in positive ion mode between (B) Cit and Cit-Gluc, (D) Gluc and Cit-Gluc and (F) Gluc and Cit.

Figure S4. KEGG enrichment analysis of DAMs between (A) Gluc and Cit-Gluc and (B) Gluc and Cit, respectively.

Figure S5. The heat map of transcription factors (Z-scored).

Table captions

Table S1. Differentially accumulated metabolites identified between Cit and Cit-Gluc sample groups.

Table S2. KEGG pathway enrichment analysis of differentially accumulated metabolites.

Table S3. The differentially accumulated metabolites in top enriched pathways.

Table S4. KEGG pathway enrichment analysis of differentially accumulated metabolites of profile 0 and profile 1.

Table S5. Summary of RNA-seq data and sequence assembly.

Table S6. Differentially accumulated metabolites in joint KEGG enrichment pathways.

Table S7. Potential transcriptional metabolic regulatory network in CCR.

Table S8. Pearson correlation coefficient of DEGs with TF or DAMs in potential transcriptional metabolic regulatory network.

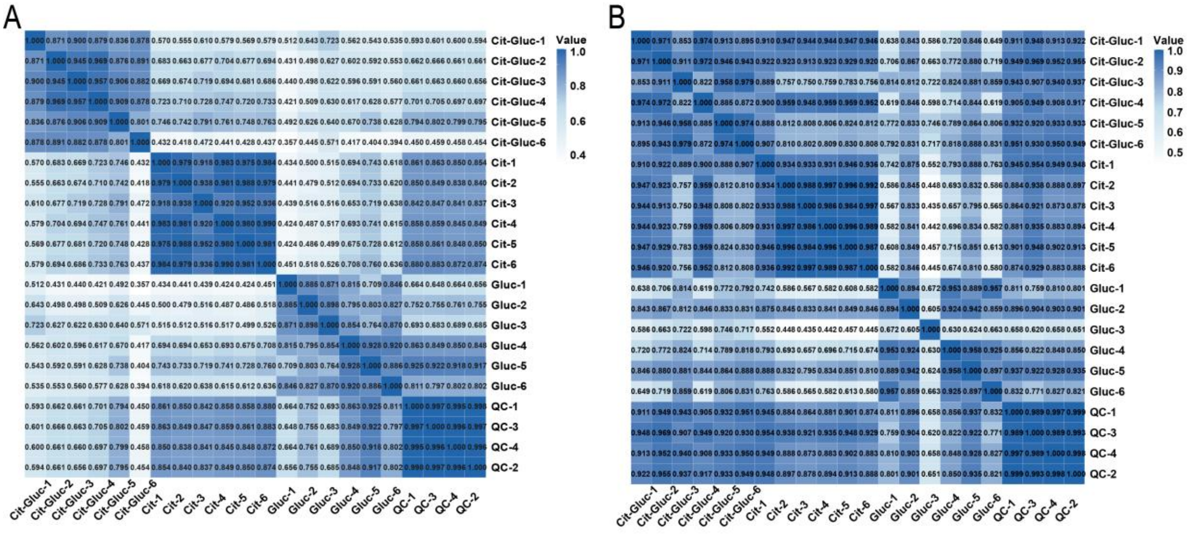

Figure S1. Pearson's correlation coefficients of different fermented samples in (A) negative ion mode and (B) positive ion mode.

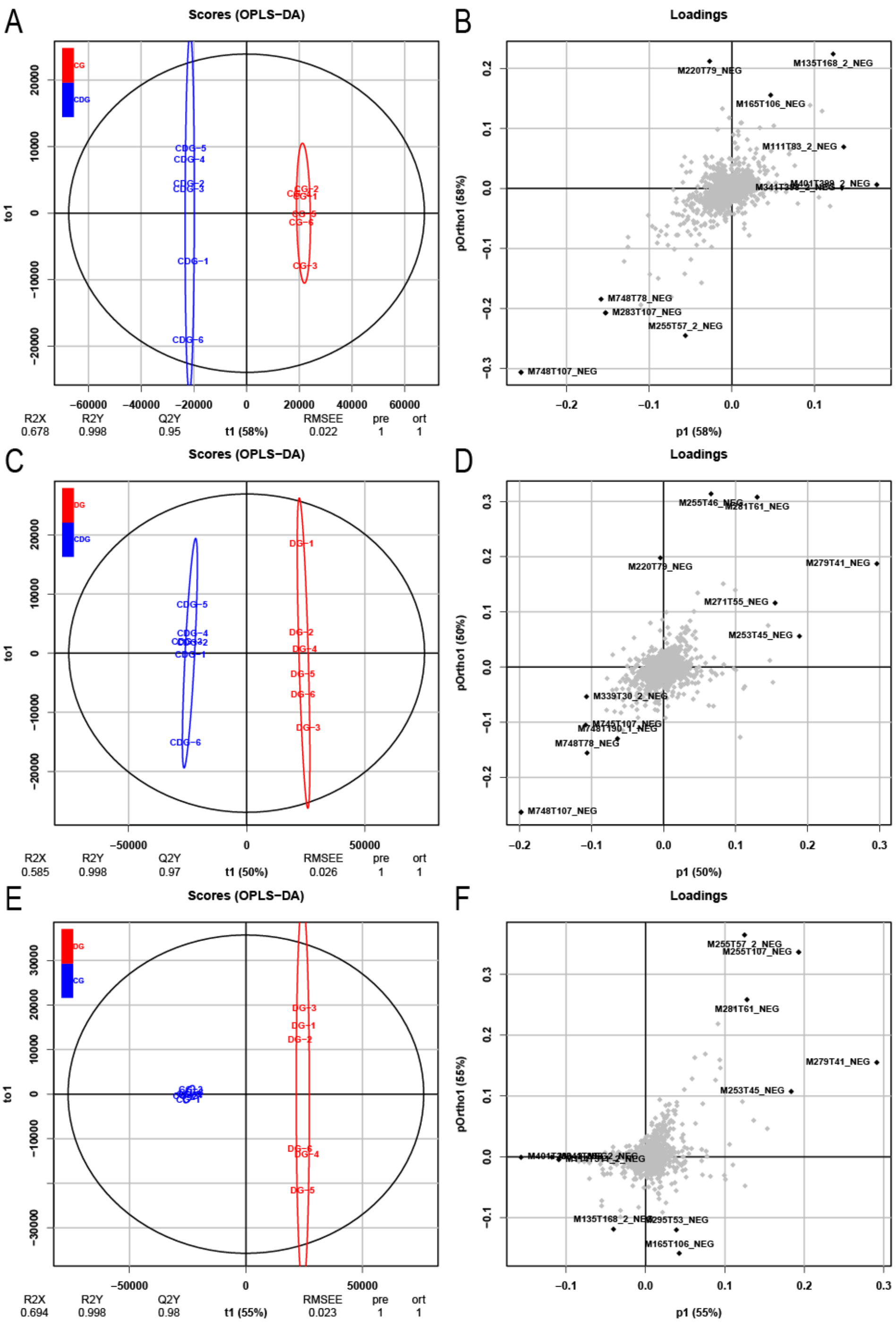

Figure S2. The OPLS-DA scores of metabolites in negative ion mode between (A) Cit and Cit Gluc, (C) Gluc and Cit-Gluc and (E) Gluc and Cit; and the OPLS-DA loading of metabolites in negative ion mode between (B) Cit and Cit-Gluc, (D) Gluc and Cit-Gluc and (F) Gluc and Cit.

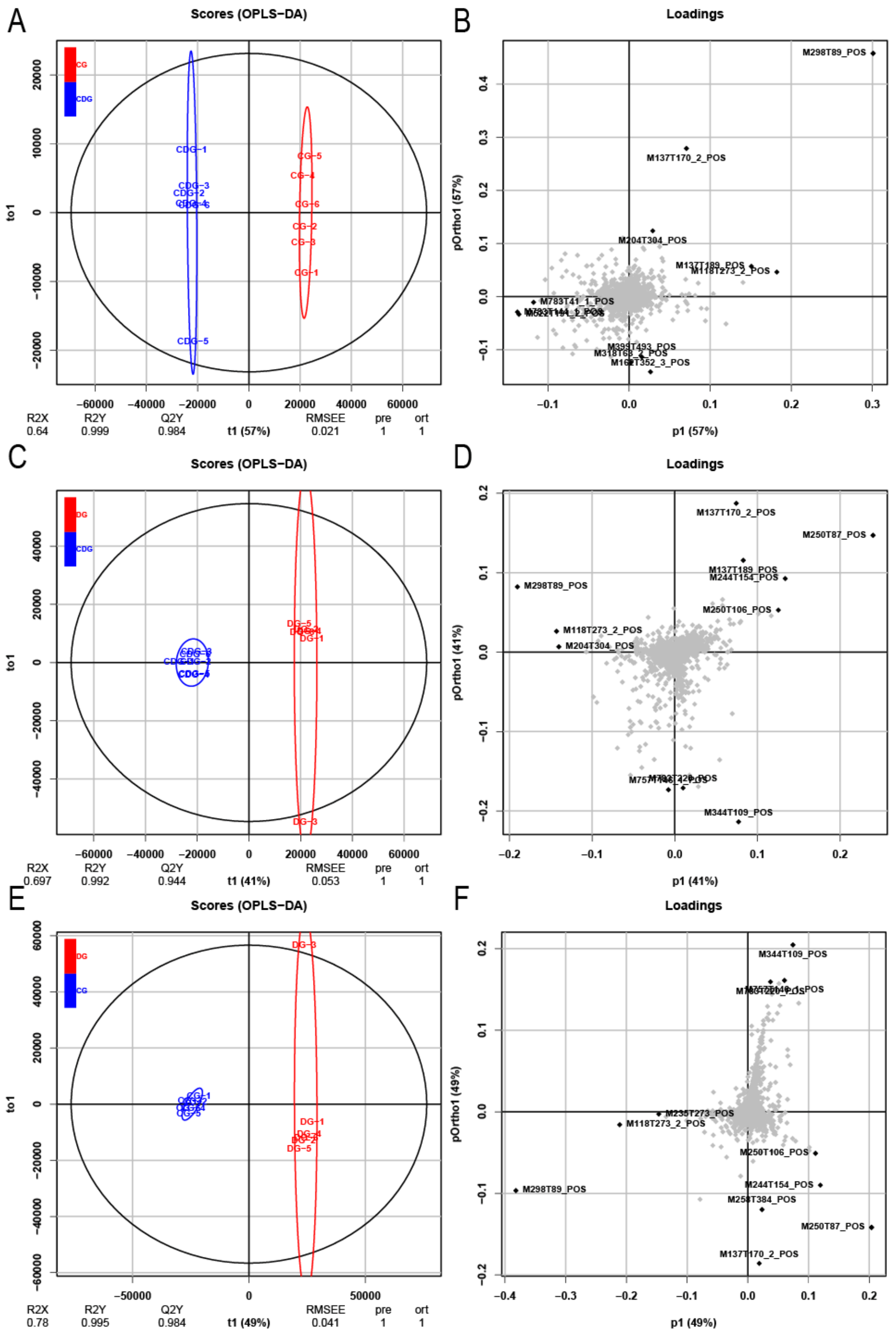

Figure S3. The OPLS-DA scores of metabolites in positive ion mode between (A) Cit and Cit Gluc, (C) Gluc and Cit-Gluc and (E) Gluc and Cit; and the OPLS-DA loading of metabolites in positive ion mode between (B) Cit and Cit-Gluc, (D) Gluc and Cit-Gluc and (F) Gluc and Cit.

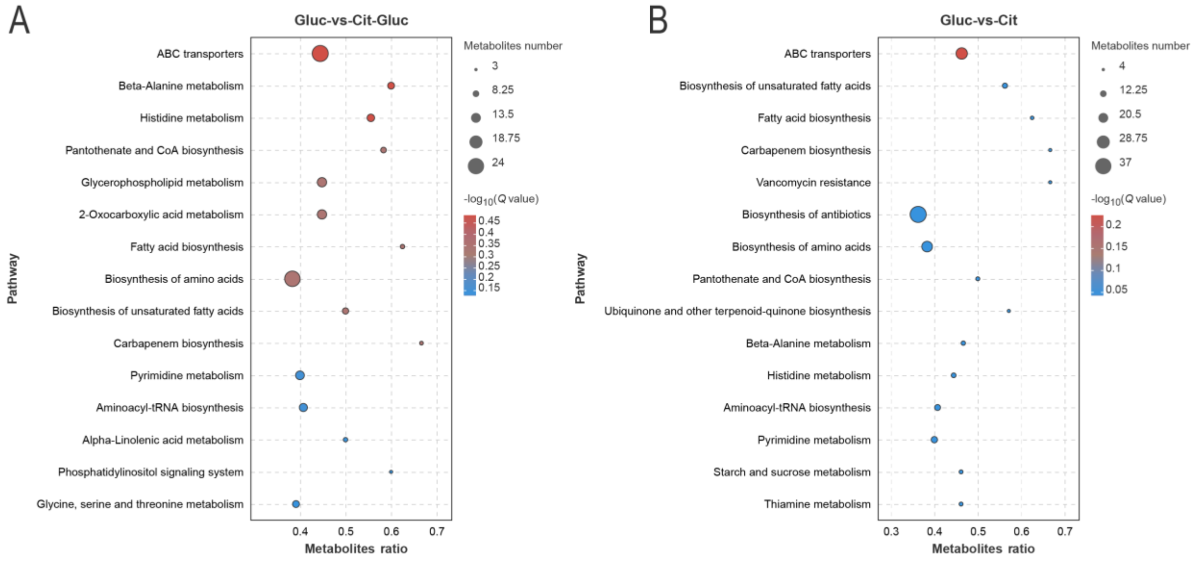

Figure S4. KEGG enrichment analysis of DAMs between (A) Gluc and Cit-Gluc and (B) Gluc and Cit, respectively.

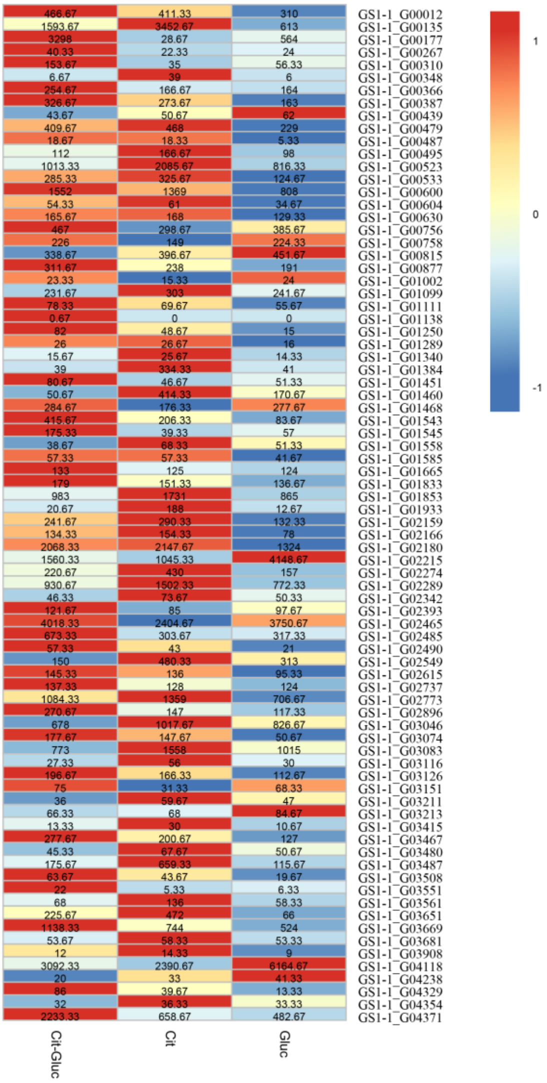

Figure S5. The heat map of transcription factors (Z-scored).
